# The outpatient experience questionnaire for child and adolescent mental health services: reliability and validity following a nationwide survey

**DOI:** 10.1186/s41687-025-00852-x

**Published:** 2025-02-18

**Authors:** Hilde Hestad Iversen, Mona Haugum, Oyvind Bjertnaes

**Affiliations:** Norwegian Institute of Public Health: Folkehelseinstituttet, PO Box 222, Skoyen, Oslo 0213 Norway

**Keywords:** Questionnaire, Patient experiences, Adolescent, Psychometrics, Reliability, Validity

## Abstract

**Background:**

The main objective was to evaluate the psychometric properties of the OutPatient Experience Questionnaire for Child and Adolescent Mental Health Services (OPEQ-CAMHS) among patients aged 16 and above, with a secondary objective of developing a parsimonious set of items and a short version of the instrument for this age group.

**Methods:**

A national pilot study was conducted with adolescents from outpatient CAMHS in Norway, testing a new digital, continuous measurement approach using a measurement instrument developed through a comprehensive methodological framework. The study assessed missing data, ceiling effects, factor structure, internal consistency, discriminant validity, and construct validity. A shorter version was derived from psychometric results.

**Results:**

The pilot study included responses from 555 (46.3%) patients. Low proportions of missing or “not applicable” responses were found for 18 of the 20 items, and all items were below the ceiling-effect criterion. Two scales emerged: “structure and process”, and “outcome”, both meeting alpha criteria. Each individual item demonstrated a stronger correlation with its expected scale than with any of the other scales. Construct validity was confirmed through associations with relevant variables expected to be associated with patient-reported experiences, including self-reported current state and well-being. The results supported a six-item short version.

**Conclusions:**

Psychometric testing confirmed data quality, internal consistency, and construct validity of OPEQ-CAMHS. The short version addresses respondent burden concerns and is now ready for broad implementation in Norwegian CAMHS and potentially in similar healthcare settings worldwide.

## Background

Patient experiences are increasingly recognized as crucial for improving healthcare quality, alongside clinical effectiveness and patient safety [1–3]. A systematic review found consistent positive associations between patient experience, safety and effectiveness across various disease areas, settings, outcome measures and study designs [1].

Mental health disorders among adolescents pose economic and public health challenges with long-lasting consequences [4], with over one in ten experiencing mental illness at any given time [5]. However, there is limited knowledge about adolescent involvement in mental healthcare, and few studies have evaluated their experiences [4, 6, 7]. Fewer studies focus on younger populations, and gaps exist in understanding the use of patient-reported experience measures (PREMs) in paediatrics [8]. Little is also known about the routine use of PREMs to improve experience and outcomes in child and adolescent mental health services (CAMHS) [9].

Young people have unique health care needs and perceptions, necessitating specific measurement of their experiences [10]. Review findings highlight eight key domains crucial to young people’s care experiences, including clinician attitudes, communication, competency, involvement, guideline-driven care, outcomes, accessibility, and age-appropriate environments [11]. In mental health services, adolescents prioritize involvement in care decisions, privacy, age-appropriate information, and a trusting relationship with professionals [9]. Staff consistency is crucial in mental health services for adolescents, as it fosters trust and continuity. Frequent staff changes can disrupt the therapeutic relationship, making it difficult for adolescents to feel secure and supported [9].

The Norwegian Institute of Public Health (NIPH) conducts research on patient-reported experiences and outcomes, aiming for a more patient-centred health system through surveys and continuous measurements across various patient populations [12, 13]. Previously, NIPH has mainly focused on adult services, leaving a gap in understanding younger populations’ experiences. However, in recent years, instruments have been developed to measure young patients’ experiences with outpatient diabetes care and CAMHS [14, 15]. The NIPH has previously conducted national parent experience surveys with outpatient CAMHS in 2006 and 2017. Starting in 2023, NIPH is implementing digital, continuous electronic measurements, aligning with modernizations for other patient groups [12, 13]. Continuous measurements offer a novel, feasible and time-effective approach by collecting large-scale data via web-based administration methods, encompassing both parent/guardian and patient experiences in CAMHS [16]. A person-centred approach involving both children and parents yields valuable insights into healthcare quality [17].

This study aims to determine the data quality, validity and internal consistency reliability of the OutPatient Experience Questionnaire for Child and Adolescent Mental Health Services (OPEQ-CAMHS) among patients 16 years and older following a nationwide pilot. The instrument is designed to measure patient-experiences among adolescents aged 12–18 years visiting CAMHS in Norway. However, challenges related to obtaining parental consent and digital access issues for guardians of 12–15-year-olds limited participation in this age group, therefore, only those aged 16 and older are included in this study. Additionally, we aimed to develop a short version of the instrument focused specifically on PREMs. The OPEQ-CAMHS was tested applying standard NIPH methodology [12, 13, 18–20].

## Methods

### Measurement instruments

The development and validation of the OPEQ-CAMHS followed NIPH’s standard methodology, including a literature review, patient interviews, expert-group consultations, and pilot testing [15]. The instrument was originally developed to assess patient experiences during treatment in CAMHS. For this study, we adapted the instrument slightly by modifying verb forms to reflect a post-treatment context rather than during ongoing treatment. Additional File 1 presents the questionnaire.

The OPEQ-CAMHS comprises 40 closed-ended items, with 28 addressing patient experiences. Two open-ended questions inquire about experiences with medications while under the care of CAMHS, and overall experiences with CAMHS. The questionnaire is divided into sections covering therapists (13 items), CAMHS cooperation with others (5 items), medication (11 items), other questions about CAMHS including the effectiveness of support in managing daily life, satisfaction with treatment, feelings of coercion regarding CAMHS attendance, and the necessity of contacting CAMHS when starting treatment (4 items), and background questions (6 items). The final question in the survey asks who completed the survey: the respondent alone, together with parents/guardians, or parents/guardians by themselves.

Most experience items use a five-point response format ranging from 1 (not at all) to 5 (to a very large extent), with an additional option for “not applicable/don’t know”. This response scale is consistently applied in NIPH surveys, facilitating comparisons over time and among different healthcare user groups [12, 18–20]. Smiley face icons illustrate most response options.

The Five-item World Health Organization Well-being Index (WHO-5) is a widely used patient-reported outcome measure (PROM) designed to assess subjective well-being [21, 22]. It has been validated for screening depression and as an outcome measure in clinical trials, demonstrating applicability across various study fields [21]. In this study, the WHO-5 was selected to measure patients’ emotional well-being, comprising five positively worded items covering cheerfulness, calmness, activity, rest, and interest [23]. Respondents rate how well each statement applies to them over the last 14 days, with scores ranging from 5 (all of the time) to 0 (at no time), resulting in a raw score ranging from 0 (absence of well-being) to 25 (maximal well-being).

Additionally, a separate module addressing medication experiences within CAMHS was included in the instrument [24], but it is not part of the generic measure.

### Data collection

The OPEQ-CAMHS was tested in a national pilot including patients at outpatient CAMHS in Norway, serving as the initial step to develop and validate new continuous measurements.

Data collection was conducted electronically between January-February 2023 and October-November 2023. Samples were obtained from the Norwegian Patient Registry (NPR), and participants were invited through Helsenorge.no, covering over 90% of Norway’s population. Inclusion criteria required patients to have ended CAMHS treatment in November 2022, August 2023, or September 2023. For children under 12, only parents were invited to complete the Parent experiences questionnaire for outpatient child and adolescent mental health services (PEQ-CAMHS Outpatients) [25].

The pilot included patients aged 12 and older. Patients under 16 required parental consent, while those 16 and older could provide their own consent. Due to access issues on the Helsenorge.no platform for guardians of 12–15-year-olds, it was necessary to invite guardians (and their children) via postal mail. As parental consent was required before inviting children aged 12–15, this limited the number of invited participants in this age group. Only 49 out of 595 eligible participants in this cohort were invited, and of these, 20 responded. Given the small number of respondents and the challenges associated with data collection in this age group, we chose to exclude the 12–15-year-olds from the current study. Therefore, this study focuses on patients 16 and older. Of 1,593 eligible patients in this age group, 1,200 were reached via Helsenorge.no (75.3%). Non-respondents received up to three SMS reminders. Due to a low response rate in the November 2022 group, a lottery incentive was introduced for the August and September 2023 groups (10 gift cards of NOK 5,000 each).

### Statistical analysis

Missing data and ceiling effects were assessed, with items exceeding > 20% of missing data and “not applicable” responses excluded from factor analysis to minimize response loss [12, 19, 20]. Ceiling effects, indicating the percentage of respondents selecting the most favourable response option, were deemed acceptable if below 50% [12, 19, 20, 26].

Exploratory factor analysis (EFA) and Item Response Theory (IRT) were applied to evaluate instrument properties. Items with a five-point scale on outpatient CAMHS experiences were analysed. EFAs were conducted using principal-axis factoring to assess the underlying factor structure of the OPEQ-CAMHS, with promax rotation selected for interpretability. Factors with eigenvalues > 1 were rotated. Items with loadings < 0.40 or cross-loadings > 0.30 were excluded. The analyses combined data-driven and theoretical approaches, separating outcome items from process and structure items, consistent with previous psychometric testing in the national survey program. Following the guidance of Kilbourne et al., mental healthcare quality measures should be validated across Donabedian’s framework, encompassing structure, process, and outcomes [27]. This conceptual foundation guided the two-step factor analysis, as we aimed to measure different components of patient experiences in alignment with the program’s design, i.e., separating outcome items from process and structure items. Scale reliability was confirmed using item-total correlation and Cronbach’s alpha, with a minimum acceptable value of 0.7 [28]. To explore the potential influence of the lottery incentive introduced in the August/September sample, we conducted separate EFAs and assessed scale reliability for the November and August/September samples. The same procedures were applied to both groups as for the full sample.

To assess discriminant validity, we examined the correlations between each item and the scale it was hypothesized to belong to, as well as its correlations with other scales. The scales were scored by first recoding the individual item responses from a 1–5 scale to a 0–100 scale. We then computed an overall score for each scale by averaging the recoded items, with the requirement that at least half of the items must have valid responses for the score to be calculated. We expected the items to show stronger correlations with their respective hypothesized scales than with other scales. Spearman’s rho was used to compute these correlations.

Construct validity evaluates how well scores from health-related patient-reported outcomes (HR-PRO) instruments align with theoretical expectations, including internal correlations and group differences, ensuring accurate measurement of the intended construct [29]. In CAMHS, factors such as involvement in treatment decisions, sufficient time for questions, and satisfaction with appointment frequency are crucial for adolescent satisfaction [30], outweighing socio-demographic and disorder severity factors. For adult psychiatric inpatients, coerced admission and treatment, along with self-perceived current state, significantly influence outcome assessments [31]. This study hypothesizes correlations between background variables, including perceived need for CAMHS contact, coercion perception regarding CAMHS initiation, overall well-being assessment, and WHO-5 scores and scale scores, analysed using Spearman’s rank correlations. We expected small to moderate positive correlations between perceived need for CAMHS contact and patient experiences, as well as between patient experiences and WHO-5 scores. Conversely, we anticipated a small to moderate negative correlation between feelings of coercion and patient experiences.

IRT analyses enhanced our understanding of item performance within the OPEQ-CAMHS, crucial for developing its short version [32]. We applied the Generalized Partial Credit Model (GPCM) for its adaptability in estimating item-discrimination and category-response parameters, focusing on “structure and process” items. The scale contained sufficient items for IRT analyses, enabling detailed insights into each item’s function across the latent construct. Key analytical aspects included item discrimination (‘a’), item difficulty or location (‘b’), and the S − χ² item-fit statistic. A higher ‘a’ value indicates greater sensitivity to changes in the latent trait, while a lower value suggests less sensitivity. The S–χ² value assesses model fit for each item, with lower values indicating better fit and reliable measurement of the intended construct [33]. However, these fit indices are sensitive to sample size, with large samples potentially yielding poor fit even for trivial discrepancies [32]. Item difficulty parameters (b1 to b4) provide critical insights into the latent trait threshold at which respondents are likely to agree with each item. Higher negative values indicate easier endorsement, while lower values indicate greater difficulty.

Our main objective was to condense the OPEQ-CAMHS to its most impactful items for a concise version, considering missing data, ceiling effects, and insights from EFA and IRT. The consistency between the shortened and full version of the “structure and process” scale was assessed using intraclass correlation coefficients (ICCs). Statistical analyses were conducted using SPSS (version 28.0.1.0) and R (version 4.0.2), with specialized analyses performed using the lavaan, semPlot, and mirt packages.

The WHO-5 measure well-being using five items scored on a five-point scale, reflecting subjective well-being over a 2-week period [21, 22]. As scales measuring health related quality of life are conventionally translated to a percentage scale from 0 (absent) to 100 (maximal), it is recommended to multiply the raw score by 4 [21]. An index was computed by summing the evaluations, following previous research and using consistent measures [34].

## Results

We received 133 responses (35.8%) from patients in November 2022. Response rates for the August and September 2023 groups were 52.1% and 49.9%, respectively. The NIPH received 555 completed questionnaires, achieving a total response rate of 46.3%. The vast majority (97.6%) were completed by the patients themselves, while a small proportion were completed either jointly with parents/guardians (2.2%) or by parents/guardians alone (0.2%). Girls comprised the majority of participants, accounting for 81.8% of the sample (n = 454) (Table 1). In terms of the age distribution, 16-year-olds made up 30.1% (n = 167), while 17-year-olds constituted 43.2% (n = 240) of the sample. Regarding perceived need for help from CAMHS, 44.4% (n = 232) felt they needed assistance “to a very large extent”, while 38.6% (n = 208) did not feel forced at all to seek help. When asked about their current well-being, 26.3% (n = 145) reported feeling “very poor” or “rather poor”. The mean WHO-5 score was 42.0 (SD = 22.8).

**Table 1:** Background variables (*n* = 555)

	*n*	%
Sex		
Male	101	18.2
Female	454	81.8
Age, years		
16	167	30.1
17	240	43.2
18	126	22.7
19	19	3.4
20	3	0.5
Did you feel that you needed contact with CAMHS?		
Not at all	30	5.7
To a small extent	34	6.5
To a moderate extent	86	16.4
To a large extent	141	27.0
To a very large extent	232	44.4
Did you feel forced by others to start going to CAMHS?		
Not at all	208	38.6
To a small extent	121	22.4
To a moderate extent	103	19.1
To a large extent	44	8.2
To a very large extent	63	11.7
Overall, how are you feeling today?		
Very poor	43	7.8
Rather poor	102	18.5
Both-and	223	40.5
Rather good	127	23.0
Very good	56	10.2
WHO-5	550	42.0/22.8 (mean/SD)

Table 2 presents the levels of missing data, responses in the “not applicable” option, mean values and ceiling effects for the 20 items related to patient experiences considered relevant for psychometric testing. Missing data ranged from 0.4% to 1.4%, and responses in the “not applicable” category varied from 0.0–38.2%. Except for two items, all OPEQ-CAMHS items had fewer than 20% missing or “not applicable” responses. The exceptions were item 16 (“Was the cooperation between CAMHS and other family members or your social circle good for you?”) (39.6%) and item 14 (“Were you able to talk to your therapist about things that were important to you when your parents/guardians were with you during your appointments?”) (20.2%). The mean score on the scale from 1 to 5, where 5 represented the best possible experience, was highest for item 1 (“Did you feel welcomed by your therapist?”) (4.08), and lowest for item 11 (“Did you and your therapist get to know each other well enough before talking about difficult subjects?”) (2.90). All items met the ceiling-effect criterion (<50% in the most-positive response option), and none were excluded from further analyses due to this criterion.

**Table 2:** Item descriptions for the OPEQ-CAMHS

		*n*	Missing (%)	Not applicable (%)	Mean*	Ceiling (%)
**Patient experiences**						
1	Did you feel welcomed by your therapist?	552	0.5	-	4.08	38.4
2	Did you get enough time with your therapist?	548	1.3	-	3.42	17.2
3	Did your therapist provide satisfactory answers if you had any questions?	531	0.4	4.0	3.50	21.5
4	Did your therapist have good suggestions for how you could both work together?	533	0.5	3.4	3.47	20.6
5	Did your therapist seem to be good at their job?**	544	0.7	1.3	3.80	34.6
6	Did you feel that your therapist listened to what you had to say?**	551	0.7	-	3.76	34.8
7	Did your therapist seem to understand how you were feeling?	541	0.7	1.8	3.29	18.1
8	Did you feel that your therapist cared about you?**	541	0.5	2.0	3.75	32.0
9	Did you feel safe with your therapist?	545	0.5	1.3	3.61	28.4
10	Were you able to talk to your therapist about things that were important to you?**	541	0.7	1.8	3.60	27.4
11	Did you and your therapist get to know each other well enough before talking about difficult subjects?	529	0.7	4.0	2.90	9.8
12	Were you able to take part in making decisions about your treatment?	515	0.9	6.3	3.29	20.2
13	Were you able to take part in making decisions about what happened during your appointments?	518	0.7	5.9	3.29	16.6
14	Were you able to talk to your therapist about things that were important to you when your parents/guardians were with you during your appointments?	543	1.3	18.9	3.14	16.9
15	Was the cooperation between CAMHS and your parents/guardians good for you?	562	0.7	16.0	3.32	18.8
16	Was the cooperation between CAMHS and other family members or your social circle good for you?	335	1.4	38.2	3.16	16.7
17	Was the cooperation between CAMHS and your school PPT (psychological counselling service), your GP, or other services good for you?	473	0.9	13.9	3.22	16.7
18	Did you feel that you were given the opportunity to decide what your therapist shared about you with others CAMHS was in contact with?	482	0.9	12.3	3.65	30.1
30	Overall, did you get the help you needed to better manage everyday life?**	531	0.7	3.6	2.98	15.4
31	Overall, did you get good help and treatment from CAMHS?**	549	1.1	0.0	3.14	16.3

*Most items were scored on a 5-point response scale ranging from 1 (“not at all”) to 5 (“to a very large extent”)

**Items finally selected for the short version of the instrument

The first EFA included the 16 items related to “structure and process”. The two “outcome” items were included in the second analysis. No items were excluded due to low factor loading (<0.40), or cross-loadings > 0.30. The EFA for “structure and process” items identified one factor explaining 63.3% of the variance (Table 3). The second EFA included the “outcome” items and identified one factor explaining 92.9% of the variance. Item-total correlations for both scales were acceptable (0.65 to 0.86), and Cronbach’s alpha values ranged from 0.92 to 0.96, meeting the criterion (0.70) (Table 3). To examine the potential impact of the lottery incentive on factor structure and scale reliability, we conducted separate analyses for the November and August/September groups. The results indicated consistent factor structures across both samples, with one factor identified for “structure and process” items and one for “outcome” items in each group (results not shown). Cronbach’s alpha values were acceptable across both samples, and the variance explained by the factors remained stable.

**Table 3:** Factor loadings and reliability statistics for the OPEQ-CAMHS

		Factor loading*	Corrected item-total correlation	Cronbach’s alpha	Cronbach’s alpha if item deleted
**Experiences with CAMHS - structure and process**				0.96	
10	Were you able to talk to your therapist about things that were important to you?	0.86	0.85		0.96
8	Did you feel that your therapist cared about you?	0.86	0.84		0.96
6	Did you feel that your therapist listened to what you had to say?	0.86	0.84		0.96
9	Did you feel safe with your therapist?	0.85	0.83		0.96
4	Did your therapist have good suggestions for how you could both work together?	0.85	0.83		0.96
5	Did your therapist seem to be good at their job?	0.85	0.82		0.96
7	Did your therapist seem to understand how you were feeling?	0.84	0.82		0.96
3	Did your therapist provide satisfactory answers if you had any questions?	0.84	0.81		0.96
1	Did you feel welcomed by your therapist?	0.81	0.79		0.96
11	Did you and your therapist get to know each other well enough before talking about difficult subjects?	0.77	0.75		0.96
13	Were you able to take part in making decisions about what happened during your appointments?	0.70	0.69		0.96
12	Were you able to take part in making decisions about your treatment?	0.67	0.66		0.96
15	Was the cooperation between CAMHS and your parents/guardians good for you?	0.67	0.66		0.96
2	Did you get enough time with your therapist?	0.67	0.66		0.96
18	Did you feel that you were given the opportunity to decide what your therapist shared about you with others CAMHS was in contact with?	0.67	0.66		0.96
17	Was the cooperation between CAMHS and your school PPT (psychological counselling service), your GP, or other services good for you?	0.66	0.65		0.96
**Experiences with CAMHS - outcome**				0.92	
30	Overall, did you get the help you needed to better manage everyday life?	0.86	0.86		-
31	Overall, did you get good help and treatment from CAMHS?	0.86	0.86		-

*Separate factor analysis for i) structure ad process items, and ii) outcome items

Table 4 shows that all items exhibited stronger correlations with their respective scale than with the other scale, with the item-to-own-scale correlation coefficients ranging from 0.66 to 0.97. All correlations were statistically significant (*p* < 0.001).

**Table 4:** Correlations between items and scales for the OPEQ-CAMHS

		Structure and process	Outcome
**Experiences with CAMHS - structure and process**			
1	Did you feel welcomed by your therapist?	**0.81**	0.65
2	Did you get enough time with your therapist?	**0.66**	0.57
3	Did your therapist provide satisfactory answers if you had any questions?	**0.85**	0.73
4	Did your therapist have good suggestions for how you could both work together?	**0.85**	0.72
5	Did your therapist seem to be good at their job?	**0.85**	0.71
6	Did you feel that your therapist listened to what you had to say?	**0.84**	0.69
7	Did your therapist seem to understand how you were feeling?	**0.84**	0.73
8	Did you feel that your therapist cared about you?	**0.84**	0.68
9	Did you feel safe with your therapist?	**0.83**	0.68
10	Were you able to talk to your therapist about things that were important to you?	**0.86**	0.70
11	Did you and your therapist get to know each other well enough before talking about difficult subjects?	**0.79**	0.62
12	Were you able to take part in making decisions about your treatment?	**0.74**	0.62
13	Were you able to take part in making decisions about what happened during your appointments?	**0.75**	0.58
15	Was the cooperation between CAMHS and your parents/guardians good for you?	**0.70**	0.63
17	Was the cooperation between CAMHS and your school PPT (psychological counselling service), your GP, or other services good for you?	**0.66**	0.57
18	Did you feel that you were given the opportunity to decide what your therapist shared about you with others CAMHS was in contact with?	**0.70**	0.50
**Experiences with CAMHS - outcome**			
30	Overall, did you get the help you needed to better manage everyday life?	0.78	**0.97**
31	Overall, did you get good help and treatment from CAMHS?	0.82	**0.96**

Correlations in bold show item to own scale correlations

Table 5 shows that all tests of construct validity were statistically significant. The correlation between patients’ perceived need for CAMHS contact and their experiences with CAMHS was significant, with stronger perceived need correlating with more positive experiences. Scale scores correlated weakly to moderately with feelings of being coerced into CAMHS and self-reported general condition, ranging from − 0.18 to 0.41. Lower coercion levels and better general condition were associated with higher scale scores. Higher WHO-5 scores, which reflect better psychological well-being, were significantly correlated with more positive patient experiences.

**Table 5:** Construct validity testing: associations between scales, background variables and responses to individual questionnaire items

	Structure and process	Outcome
Did you feel that you needed contact with CAMHS?	0.23***	0.27***
Did you feel forced by others to start going to CAMHS?	−0.23***	−0.18***
Overall, how are you feeling today?	0.33***	0.41***
WHO-5	0.33***	0.40***

***p < 0.001; **p < 0.01; *p < 0.05; ns, not significant. Data are represented as Spearman’s rank correlation coefficients

In the IRT analysis of the OPEQ-CAMHS “structure and process” scale, we examined item performance, discrimination, difficulty levels, and sensitivity to patient experiences. Key findings highlight variations in item fit, discrimination parameters, and difficulty thresholds, as well as identification of items that are highly sensitive to differences in patient experiences. It is important to note that this analysis is applied to the “structure and process” scale due to the limited number of items in the “outcome” scale.

Table 6 displays the IRT analysis parameters including discrimination (a), difficulty thresholds (b1–b4), and item fit (S-χ2 and p-values) for the OPEQ-CAMHS “structure and process” scale. The S − χ^2^ varied from 32.21 to 93.79, with all items showing satisfactory performance based on p-values. Item 15 (“Was the cooperation between CAMHS and your parents/guardians good for you?”) exhibited the highest S–χ² value at 93.79, but the corresponding p-value suggest an adequate fit. Item 3 (“Did your therapist provide satisfactory answers if you had any questions?”) had the lowest S–χ² value of 32.21, indicating a good fit to the model.

**Table 6:** Parameter estimates derived from IRT analysis of the OPEQ-CAMHS, Experiences with CAMHS - structure and process

		*a*	b1	b2	b3	b4	S–${{{\chi }}^2}$	*p*
**Experiences with CAMHS - structure and process**								
1	Did you feel welcomed by your therapist?	2.87	−1.97	−1.60	−0.88	0.26	37.92	0.382
2	Did you get enough time with your therapist?	1.10	−1.94	−1.10	−0.25	1.16	60.69	0.594
3	Did your therapist provide satisfactory answers if you had any questions?	2.63	−1.72	−1.13	−0.12	0.81	32.21	0.886
4	Did your therapist have good suggestions for how you could both work together?	2.76	−1.72	−0.99	−0.16	0.84	61.15	0.055
5	Did your therapist seem to be good at their job?	3.03	−1.48	−1.27	−0.51	0.28	57.58	0.099
6	Did you feel that your therapist listened to what you had to say?	2.83	−1.38	−1.16	−0.46	0.29	47.17	0.384
7	Did your therapist seem to understand how you were feeling?	2.37	−1.18	−0.92	−0.01	0.88	47.43	0.616
8	Did you feel that your therapist cared about you?	3.21	−1.57	−1.12	−0.47	0.38	49.76	0.255
9	Did you feel safe with your therapist?	2.77	−1.43	−1.14	−0.38	0.53	48.05	0.275
10	Were you able to talk to your therapist about things that were important to you?	2.97	−1.64	−1.06	−0.23	0.53	38.80	0.524
11	Did you and your therapist get to know each other well enough before talking about difficult subjects?	1.43	−0.85	−0.57	0.39	1.43	63.01	0.616
12	Were you able to take part in making decisions about your treatment?	0.92	−1.21	−0.91	−0.05	0.98	76.18	0.689
13	Were you able to take part in making decisions about what happened during your appointments?	1.15	−1.72	−0.83	0.07	1.18	83.54	0.097
15	Was the cooperation between CAMHS and your parents/guardians good for you?	1.00	−1.28	−1.27	0.13	1.01	93.79	0.107
17	Was the cooperation between CAMHS and your school PPT (psychological counselling service), your GP, or other services good for you?	0.93	−1.39	−1.07	0.30	1.03	66.95	0.886
18	Did you feel that you were given the opportunity to decide what your therapist shared about you with others CAMHS was in contact with?	1.00	−1.17	−1.17	−0.77	0.66	85.24	0.412
		0.92 to 3.21	–1.97 to –0.85	–1.60 to –0.57	–0.88 to 0.39	0.26 to 1.43		

*a*: discrimination; b1–b4: thresholds. S–${{{\chi }}^2}$ represents item fit statistics, with P values < 0.05 indicating lack of fit

The ‘a’ values, reflecting an item’s discrimination ability, ranged from 0.92 to 3.21. Item 8 (“Did you feel that your therapist cared about you?”) displayed the highest ‘a’ value of 3.21, indicating high sensitivity to patient experience variation, closely followed by item 5 (“Did your therapist seem to be good at their job?”) with 3.03. Conversely, items 12 (“Were you able to take part in making decisions about your treatment?”) and 17 (“Was the cooperation between CAMHS and your school PPT (psychological counselling service), your GP, or other services good for you?”), had the lowest ‘a’ values (0.92 and 0.93 respectively), indicating lower discrimination effectiveness.

The difficulty parameters, represented by b1 to b4, reveal the threshold at which respondents are inclined to agree with various questionnaire items (Table 6). The b1 range from − 1.97 to − 0.85 indicates varied difficulty levels across items. Similarly, for b2, b3, and b4, the ranges were − 1.60 to − 0.57, − 0.88 to 0.39, and 0.26 to 1.43, respectively. The variation in these difficulty parameters suggests that the questionnaire captures a diverse range of experiences and could be sensitive to varying levels of patient experiences.

**Fig. 1:**
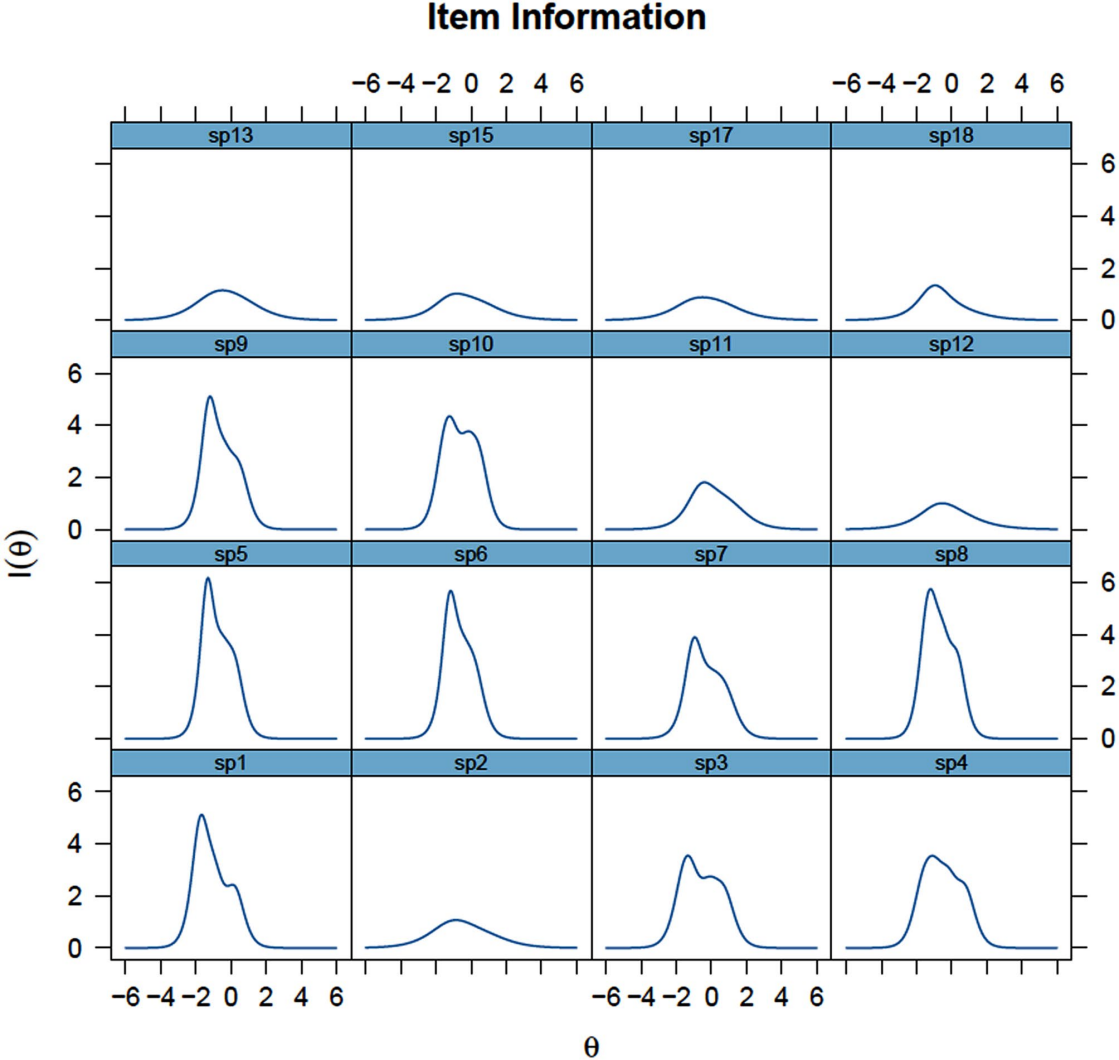
Item information functions for the OPEQ-CAMHS, Experiences with CAMHS - structure and process

**Fig. 2:**
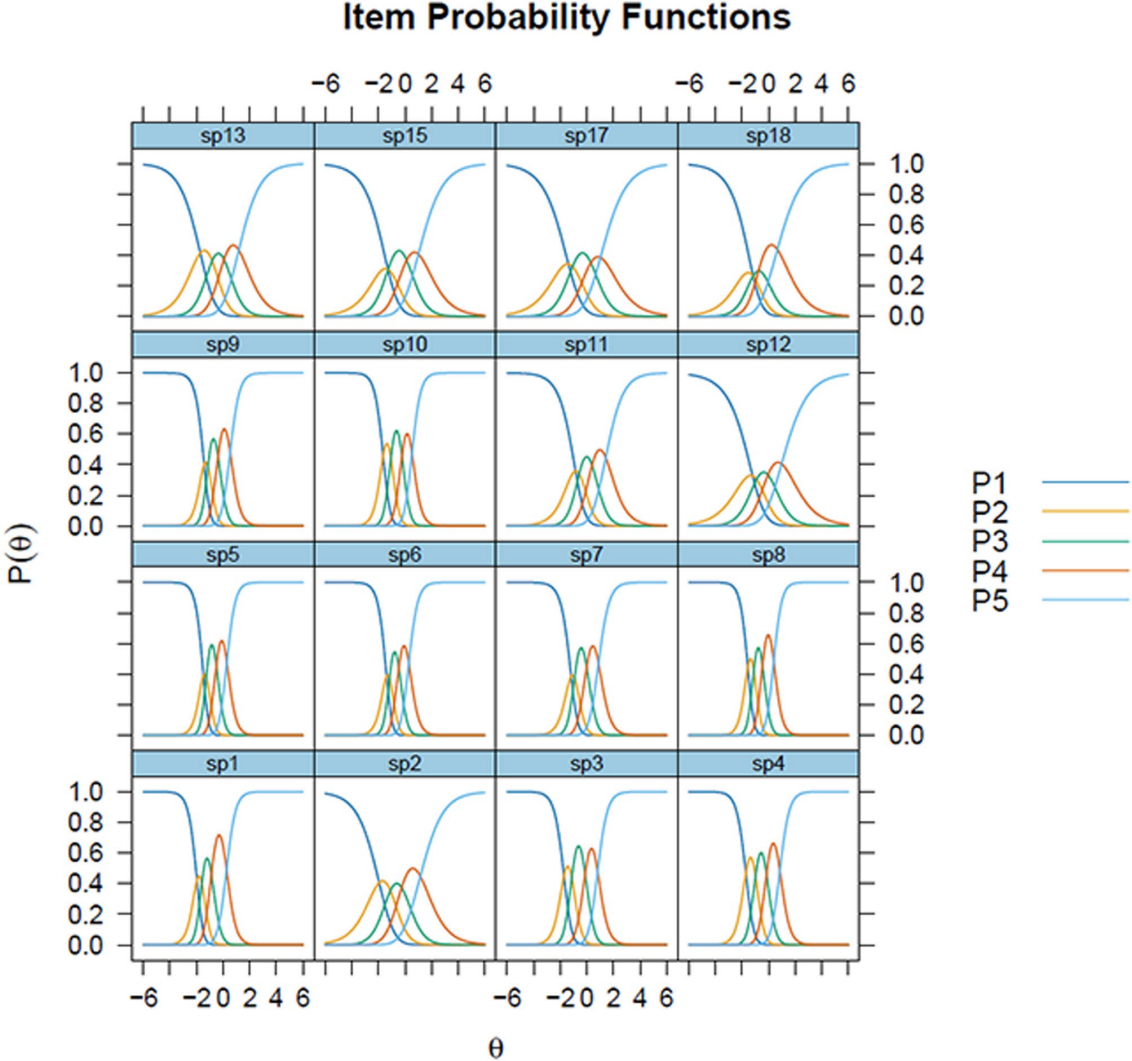
Categorical response curves for the OPEQ-CAMHS, Experiences with CAMHS - structure and process

Figures 1 and 2 illustrate the curves and response patterns. Items 1, 5, 6 (“Did you feel that your therapist listened to what you had to say?”) 8, 9 (“Did you feel safe with your therapist?”) and 10 (“Were you able to talk to your therapist about things that were important to you?”) demonstrates steep curves, indicating heightened sensitivity to changes in the latent trait level. Conversely, items 2 (“Did you get enough time with your therapist?”), 12, 13 (“Were you able to take part in making decisions about what happened during your appointments?”), 15 and 17 display flatter curves, suggesting they are less sensitive to changes in the trait level, providing consistent information across a wider range of the trait (Fig. 1). Additionally, the categorical response curves (CRCs) in Fig. 2 highlight questionable values for the second response category, particularly for items 15 and 18 (“Did you feel that you were given the opportunity to decide what your therapist shared about you with others CAMHS was in contact with?”).

Four items were selected for the abbreviated version after comprehensive evaluation of their psychometric properties.

The EFA of the “structure and process” scale revealed high factor loadings for several items, notably items 10, 8, and 6 (Table 3). Additionally, IRT analysis informed our selection, with items 3, 1, and 10 showing the best fit according to the S − χ2 statistic, and items 8, 5, and 10 demonstrating the strongest discriminative ability (Table 6). Items 1, 5, 6, 8, 9, and 10 outperformed others in terms of ideal CRCs.

Items 5, 6, 8, and 10 were chosen, covering key themes such as therapist competence, patient’s sense of being heard and cared for, and the opportunity to discuss significant matters. The ICC comparing the full 16-item scale with the 4-item short form, was high at 0.97 (*p* < 0.001), indicating strong consistency.

To sum up, item reduction was conducted based on psychometric properties evaluated through EFA and IRT analysis. The process included assessing factor loadings, cross-loadings, and item-total correlations. No items were excluded solely based on low factor loadings. After careful evaluation of all properties and the item fit statistics derived from the IRT model, four items of the “structure and process” scale along with both “outcome” scale items were ultimately selected for the abbreviated version of the instrument.

## Discussion

This study aimed to evaluate the psychometric properties of the OPEQ-CAMHS through a nationwide pilot of continuous electronic measurements in Norway during 2022 and 2023. Additionally, it sought to provide insights into item performance and develop a shorter version of the instrument.

The psychometric testing confirmed good data quality and internal consistency. The OPEQ-CAMHS consists of two scales based on both empirical and theoretical assumptions, covering evaluations of “structure and process”, and “outcome”. The content of the scales aligns with key aspects identified in reviews of PREMs in mental health care, highlighting interpersonal, trusting relationships, patient involvement, psychological care, access, care coordination, information, and outcomes [4, 9, 11, 35–38].

The results revealed a stable and interpretable scale structure, with low rates of omitted answers and few responses in the “not applicable” option, indicating the relevance of the questions to most patients. Patient involvement in the instrument’s development, including identifying critical aspects of outpatient care, ensured that it addresses a broad range of specific domains relevant for service improvement [15].

The findings demonstrate that each item is closely associated with its intended construct, reflecting accurate measurement. Patient experiences correlate with factors such as perceived need for CAMHS contact, coercion perceptions, current condition, and psychological well-being measured by the WHO-5. Higher satisfaction levels are associated with lower coercion and better health, emphasizing the importance of addressing individual patient needs, involving patients in treatment decisions, and attending to overall psychological well-being to enhance care quality. Prior research and theoretical frameworks offered limited hypotheses, but these findings align with earlier studies, suggesting that patient experiences in CAMHS are significantly influenced by subjective perceptions and psychosocial contexts rather than solely clinical or demographic factors [30, 31, 39]. Given the limited number of studies, a comprehensive examination of factors such as treatment modalities, personal perceptions, communication preferences, and the effects of interventions is necessary to better understand patient experiences in CAMHS. Despite increased PROMs use in child mental health, there remain inconsistencies in the measures used, highlighting the need for consensus on appropriate measures [40]. This reflects a broader challenge in the field, as there is currently no consensus on the most appropriate or standard PROMs for this population. Higher WHO-5 scores correlate with improved patient experiences, supporting its criterion validity. Further evaluation, including changes from baseline scores, is warranted to assess the relevance of subjective well-being in evaluating care quality in this setting.

Moreover, the application of IRT enriched our understanding of item performance, identifying variations in discrimination and difficulty among items. This guided selection for a shorter version of the instrument, maintaining strong psychometric properties. The national reference group for the project (representatives from patient organisations and clinicians from CAMHS) emphasized the need for shorter versions for children and adolescents. The objective of refining the scale was to develop a short version capable of effectively capturing variations and temporal changes among different groups. We carefully evaluated items from the “structure and process” scale, considering psychometric properties, missing response rates, and ceiling effects. Four items were chosen for inclusion in the abbreviated version, along with both “outcome” scale items. The abbreviated OPEQ-CAMHS comprises six items, addressing perceived outcomes, therapist competence, feeling listened to, discussing important matters, and feeling cared for. Our findings show that the instrument can be effectively reduced from its original 18-item format to a concise 6-item version, crucial for minimizing respondent burden while maintaining precision. Depending on the purpose of the study and the setting in which the measurement is conducted, there may be varying needs for including background variables or medication-related questions in addition to the 6-item PREMs version.

Furthermore, IRT results showed that the response scale for some items probably could be reduced to 3- or 4-levels. However, the 5-point scale is standard for all patient groups, so changing it for some of the questions would deteriorate the possibility for cross-item/scale comparisons and cross-patient comparisons in quality evaluation and improvement work. Also, the cognitive interviews in the development project showed that the five-point Likert scale functioned well, with most participants finding it easy to use and suitable for their needs. Having several different response scales in the questionnaire could also be more cognitively challenging for respondents. However, as the standard number of response categories for adults not necessarily suits younger patients, which was indicated by the IRT findings in the present study, we point to the need for more research on response formats for adolescents.

Notably, combining of EFA and IRT yielded valuable and distinct insights into OPEQ-CAMHS performance. The benefits of the shortened questionnaire include potentially improved response rates and reduced respondent burden, and its brevity allows adaptability for diverse populations and compatible with other assessments, enhancing versatility in various research and healthcare settings.

The nationwide pilot of continuous digital measurement supports the future integration of patient-reported experiences into national healthcare quality monitoring and improvement efforts. It provides a unique opportunity for standardized and research-based measurement of all patients discharged from CAMHS in Norway. The high response rate among this young patient group is promising, given the challenges of reaching and motivating them to respond to mental health surveys [41]. While around 36% responded in the November 2022 group, around 50% responded in the other groups after including a lottery incentive. A high response rate secure feedback from a substantial proportion of all patients using CAMHS about their health care experiences, self-reported mental health and well-being. Positive experiences correlated with better adherence to preventive and treatment processes, improved clinical outcomes, enhanced patient safety, and reduced healthcare utilization [2]. This underlines the importance of measuring, monitoring and improving patients’ experiences. The OPEQ-CAMHS is now ready for large-scale implementation in Norwegian CAMHS, with potential applicability to similar healthcare systems in other countries, albeit with adjustments to technical and legal considerations.

Published evidence on adolescents’ participation in surveys about their mental healthcare experiences and outcomes is limited. Future research should aim to improve both the quality and quantity of survey data to better understand and assess adolescents’ healthcare experiences and outcomes. Our study emphasizes the potential and importance of involving adolescents in providing feedback on healthcare issues, identifying problem areas, and suggesting improvement priorities. Adolescents value being included in discussions about their mental health treatment and decision-making processes [9].

The OPEQ-CAMHS provides actionable feedback for outpatient departments, aiding in performance monitoring and identifying areas for patient-centred quality improvement. While adolescent feedback is crucial, input from parents, who are integral to treatment, is also valuable. However, for legal reasons, patients 16 years or older must first consent to involving parents. This is an avenue for further development and research.

### Strengths and limitations

This study focused on determining the data quality, validity and internal consistency reliability of the newly developed OPEQ-CAMHS questionnaire, demonstrating its reliability and feasibility for continuous electronic surveys in CAMHS.

Web-based surveys offer cost-effective advantages. Furthermore, to enhance the study’s credibility, the survey was administered by an impartial third-party entity, the NIPH, unaffiliated with healthcare provision. Exclusions based on non-registration at Helsenorge.no pose a limitation, warranting initiatives for improved coverage and research on differences between covered and non-covered patients, and follow-up studies of non-covered patients with other modes (postal, onsite, telephone etc.).

While the abbreviated questionnaire may lack comprehensiveness, it suits scenarios prioritizing respondent burden minimization. Depending on the specific context and objectives, researchers and healthcare practitioners may find it necessary to employ either the complete questionnaire or selectively incorporate items as needed [42].

This study investigates the experiences of patients within the cohort aged 16 and older. However, the OPEQ-CAMHS instrument was also developed with younger patients in mind. It has been tested for individuals aged 12–18 through qualitative interviews to identify key aspects of care, cognitive interviews, and a pilot study [15]. Future research should include psychometric evaluations for patients aged 12–15, potentially adapting the scale with additional items for younger children, thereby expanding the instrument’s applicability across a broader age range. To reach patients 12–15 years, a tailored strategy is required, where parental consent is necessary.

## Conclusions

In conclusion, the OPEQ-CAMHS demonstrates satisfactory internal consistency, reliability, and validity, making it suitable for large-scale implementation in Norwegian CAMHS. The abbreviated version is valuable for reducing respondent workload. When engaged in continuous measurements, it’s crucial to recognize the validation process as an ongoing endeavour rather than a one-time procedure.

Psychometrically, the OPEQ-CAMHS shows robust characteristics, with EFA supporting a unidimensional structure for both the structure-process and outcome items as well as satisfactory internal consistency and construct validity.

This study contributes valuable insights to the literature on patient experiences in CAMHS, providing a rigorous appraisal of a tool for assessment and valuable support to both healthcare practitioners and researchers in their pursuit of patient-centred care within the realm of child and adolescent psychiatry. The questionnaire and data collection approach are adaptable for similar healthcare systems in other countries.

Further studies may benefit from diversifying the sample and incorporating longitudinal data, facilitating a deeper exploration of the nuanced experiences and perceptions of young individuals’ experiences in mental health care settings.

## Data Availability

The data sets generated and/or analysed during the current study are not publicly available due to the need to protect personal data but are available from the corresponding author on reasonable request.

## Abbreviations

PREM: Patient-reported experience measure
CAMHS: Child and adolescent mental health services
NIPH: Norwegian Institute of Public Health
PEQ-CAMHS Outpatients: Parent experiences questionnaire for outpatient child and adolescent mental health services
OPEQ-CAMHS: OutPatient Experience Questionnaire for Child and Adolescent Mental Health Services
WHO-5: The Five-item World Health Organization Well-being Index
PROM: Patient-reported outcome measure
NPR: Norwegian Patient Registry
EFA: Exploratory factor analysis
IRT: Item response theory
HR-PRO: Health-related patient-reported outcomes
GPCM: Generalized partial credit model
ICC: Intraclass correlation coefficient
CRC: Categorical response curves
DPIA: Data Protection Impact Assessment

## References

[1] Doyle C, Lennox L, Bell D (2013) A systematic review of evidence on the links between patient experience and clinical safety and effectiveness. BMJ Open 3(1):e001570. 10.1136/bmjopen-2012-001570 23293244 10.1136/bmjopen-2012-001570 PMC3549241

[2] Anhang Price R, Elliott MN, Zaslavsky AM, Hays RD, Lehrman WG, Rybowski L, Edgman-Levitan S, Cleary PD (2014) Examining the role of patient experience surveys in measuring health care quality. Med Care Res Rev 71(5):522–554. 10.1177/1077558714541480 25027409 10.1177/1077558714541480 PMC4349195

[3] Gleeson H, Calderon A, Swami V, Deighton J, Wolpert M, Edbrooke-Childs J (2016) Systematic review of approaches to using patient experience data for quality improvement in healthcare settings. BMJ Open 6(8):e011907. 10.1136/bmjopen-2016-011907 27531733 10.1136/bmjopen-2016-011907 PMC5013495

[4] Viksveen P, Bjønness SE, Cardenas NE, Game JR, Berg SH, Salamonsen A, Storm M, Aase K (2022) User involvement in adolescents’ mental healthcare: a systematic review. Eur Child Adolesc Psychiatry 31(11):1765–1788. doi:10.1007/s00787-021-01818-2 34089383 10.1007/s00787-021-01818-2 PMC9666298

[5] Kaushik A, Kostaki E, Kyriakopoulos M (2016) The stigma of mental illness in children and adolescents: a systematic review. Psychiatry Res 243:469–494. 10.1016/j.psychres.2016.04.042 27517643 10.1016/j.psychres.2016.04.042

[6] Patel V, Flisher AJ, Hetrick S, McGorry P (2007) Mental health of young people: a global public-health challenge. Lancet 369(9569):1302–1313. 10.1016/S0140-6736(07)60368-7 17434406 10.1016/S0140-6736(07)60368-7

[7] Oruche UM, Downs S, Holloway E, Draucker C, Aalsma M (2014) Barriers and facilitators to treatment participation by adolescents in a community mental health clinic. J Psych Mental Health Nursing 21(3):241–248. 10.1111/jpm.12076 10.1111/jpm.12076 23682756

[8] Bele S, Teela L, Zhang M, Rabi S, Ahmed S, van Oers HA, Gibbons E, Dunnewold N, Haverman L, Santana M (2021) Use of patient-reported experience measures in pediatric care: a systematic review. Front Ped 9. www.frontiersin.org/articles/10.3389/fped.2021.753536 10.3389/fped.2021.753536 PMC8721567 34988035

[9] Coyne I, McNamara N, Healy M, Gower C, Sarkar M, McNicholas F (2015) Adolescents’ and parents’ views of child and adolescent mental health services (CAMHS) in Ireland. J Psych Mental Health Nursing 22(8):561–569. 10.1111/jpm.12215 10.1111/jpm.12215 25977175

[10] Clark J, MacLennan E (2023) Measuring experience of inpatient child and adolescent mental health services (CAMHS). Int J Environ Res Public Health 20(11):5940. 10.3390%ijerph20115940 37297544 10.3390/ijerph20115940 PMC10252505

[11] Ambresin A-E, Bennett K, Patton GC, Sanci LA, Sawyer SM (2013) Assessment of youth-friendly health care: a systematic review of indicators drawn from young people’s perspectives. J Adolesc Health 52(6):670–681. 10.1016/j.jadohealth.2012.12.014 23701887 10.1016/j.jadohealth.2012.12.014

[12] Iversen HH, Haugum M, Bjertnaes O (2022) Reliability and validity of the psychiatric inpatient patient experience questionnaire – continuous electronic measurement (PIPEQ-CEM). BMC Health Serv Res 22(1):897. 10.1186/s12913-022-08307-5 35821137 10.1186/s12913-022-08307-5 PMC9275271

[13] Iversen HH, Haugum M, Ellingsen-Dalskau LH, Bjertnaes O (2024) Reliability and validity of the Patient Experiences Questionnaire for Interdisciplinary Treatment for Substance Dependence – continuous Electronic Measurement (PEQ-ITSD – CEM). BMC Health Serv Res 24(1):26. 10.1186/s12913-023-10506-7 38178069 10.1186/s12913-023-10506-7 PMC10768463

[14] Iversen HH, Bjertnaes O, Helland Y, Skrivarhaug T (2019) The adolescent patient experiences of diabetes care questionnaire (APEQ-DC): reliability and validity in a study based on data from the Norwegian childhood diabetes registry. Patient Rel Outcome Meas 10:405–416. 10.2147/PROM.S232166 10.2147/PROM.S232166 PMC6938190 31920415

[15] Haugum M, Danielsen K, Iversen HH (2019) Development of a questionnaire to measure children’s and adolescents’ experiences with outpatient child and adolescent mental health services. Report in Norwegian. PasOpp-report 2−2019. Oslo: Norwegian Institute of Public Health. www.fhi.no/globalassets/bilder/rapporter-og-trykksaker/2019/utvikling-av-sporreskjema-for-a-male-barn-og-unges-erfaringer-med-bup-pasopp-rapport-2019.pdf

[16] Edbrooke-Childs J, Jacob J, Law D, Deighton J, Wolpert M (2015) Interpreting standardized and idiographic outcome measures in CAMHS: what does change mean and how does it relate to functioning and experience? Child Adolescent Mental Health 20(3):142–148. 10.1111/camh.12107 32680398 10.1111/camh.12107

[17] Gondek D, Edbrooke-Childs J, Velikonja T, Chapman L, Saunders F, Hayes D, Wolpert M (2017) Facilitators and barriers to person-centred care in child and young people mental health services: a systematic review. Clin Psychol Psychother 24(4):870–886. 10.1002/cpp.2052 27910173 10.1002/cpp.2052

[18] Bjertnæs Ø, Iversen HH, Norman R, Valderas JM (2023) Web-based public ratings of general practitioners in Norway: validation study. JMIR Format Res 7:e38932. 10.2196/38932 10.2196/38932 PMC10131642 36930207

[19] Haugum M, Iversen HH, Bjertnaes O, Lindahl AK (2017) Patient experiences questionnaire for interdisciplinary treatment for substance dependence (PEQ-ITSD): reliability and validity following a national survey in Norway. BMC Psych 17(1):73. 10.1186/s12888-017-1242-1 10.1186/s12888-017-1242-1 PMC5319076 28219361

[20] Bjertnæs OA, Iversen HH, Kjøllesdal JG (2015) PIPEQ-OS: an instrument for on-site measurements of the experiences of inpatients at psychiatric institutions. BMC Psych 15:9. 10.1186/s12888-015-0621-8 10.1186/s12888-015-0621-8 PMC4596307 26444263

[21] Topp CW, Østergaard SD, Søndergaard S, Bech P (2015) The WHO-5 well-being index: a systematic review of the literature. Psychother Psychosom 84(3):167–176. 10.1159/000376585 25831962 10.1159/000376585

[22] Lara-Cabrera ML, Bjørkly S, De Las Cuevas C, Pedersen SA, Mundal IP (2020) Psychometric properties of the five-item World Health Organization well-being index used in mental health services: protocol for a systematic review. J Adv Nurs 76(9):2426–2433. 10.1111/jan.14445 32510656 10.1111/jan.14445

[23] Bech P, Gudex C, Staehr Johansen K (2010) The WHO (Ten) Well-Being Index: validation in Diabetes. Psychother Psychosom 65(4):183–190. 10.1159/000289073 10.1159/000289073 8843498

[24] Steiro A, Skudal KE, Iversen HH, Holmboe O (2023) Development of questions to measure children’s and adolescents’ and parents’ experiences with medication in outpatient child and adolescent mental health services (CAMHS). PasOpp-report 979−2023. Oslo: Norwegian Institute of Public Health. www.fhi.no/contentassets/99f47b687bfb4f538a37540a66cb9908/utvikling-av-sporsmal-for-a-male-barn-og-unges-og-foresattes-erfaringer-med-legemidler-i-barne–og-ungdomspsykiatriske-poliklinikker-bup-pasopp-2023.pdf

[25] Garratt AM, Bjertnaes OA, Holmboe O, Hanssen-Bauer K (2011) Parent experiences questionnaire for outpatient child and adolescent mental health services (PEQ-CAMHS Outpatients): reliability and validity following a national survey. Child Adolescent Psych Mental Health 5(1):18. 10.1186/1753-2000-5-18 10.1186/1753-2000-5-18 PMC3120777 21600010

[26] Ruiz MA, Pardo A, Rejas J, Soto J, Villasante F, Aranguren JL (2008) Development and validation of the “Treatment Satisfaction with Medicines Questionnaire” (SATMED-Q). Value Health 11(5):913–926. 10.1111/j.1524-4733.2008.00323.x 18494753 10.1111/j.1524-4733.2008.00323.x

[27] Kilbourne AM, Beck K, Spaeth-Rublee B, Ramanuj P, O’Brien RW, Tomoyasu N, Pincus HA (2018) Measuring and improving the quality of mental health care: a global perspective. World Psych 17:30–38 10.1002/wps.20482 PMC5775149 29352529

[28] Streiner DL, Norman GR, Cairney J (2015) Health measurement scales: a practical guide to their development and use. Oxford University Press. 10.1093/med/9780199685219.001.0001

[29] Mokkink LB, Terwee CB, Knol DL, Stratford PW, Alonso J, Patrick DL, Bouter LM, de Vet HCW (2010) The COSMIN checklist for evaluating the methodological quality of studies on measurement properties: a clarification of its content. BMC Med Res Method 10(1):22. 10.1186/1471-2288-10-22 10.1186/1471-2288-10-22 PMC2848183 20298572

[30] Kapp C, Perlini T, Jeanneret T, Stéphan P, Rojas-Urrego A, Macias M, Halfon O, Holzer L, Urben S (2017) Identifying the determinants of perceived quality in outpatient child and adolescent mental health services from the perspectives of parents and patients. Eur Child Adolesc Psychiatry 26(10):1269–1277. 10.1007/s00787-017-0985-z 28382545 10.1007/s00787-017-0985-z

[31] Bjertnaes O, Iversen HH (2018) Inpatients’ assessment of outcome at psychiatric institutions: an analysis of predictors following a national cross-sectional survey in Norway. BMJ Open 8(12):e023587. 10.1136/bmjopen-2018-023587 30530585 10.1136/bmjopen-2018-023587 PMC6303571

[32] Nguyen TH, Han HR, Kim MT, Chan KS (2014) An introduction to item response theory for patient-reported outcome measurement. Patient 7(1):23–35. 10.1007/s40271-013-0041-0 24403095 10.1007/s40271-013-0041-0 PMC4520411

[33] Brown TA (2015) Confirmatory factor analysis for applied research. 2nd edn. The Guilford Press

[34] Organisation for Economic Co-operation and Development (OECD) (2013) OECD guidelines on measuring subjective well-being. OECD, Paris. Available from: www.oecd.org/wise/oecd-guidelines-on-measuring-subjective-well-being-9789264191655-en.htm 24600748

[35] Fernandes S, Fond G, Zendjidjian XY, Baumstarck K, Lançon C, Berna F, Schurhoff F, Aouizerate B, Henry C, Etain B, Samalin L, Leboyer M, Llorca P-M, Coldefy M, Auquier P, Boyer L, French PG (2020) Measuring the patient experience of mental health care: a systematic and critical review of patient-reported experience measures. Patient Preference Adherence 14:2147–2161. 10.2147/PPA.S255264 33192054 10.2147/PPA.S255264 PMC7653683

[36] Bhui K, Chadburn G, Crepaz-Keay D, Fenton S-J, Griffiths F, Larkin M, Mockford C, Newton E, Staniszewska S, Weich S (2019) Experiences of in-patient mental health services: systematic review. Br J Psychiatry 214(6):329–338. 10.1192/bjp.2019.22 30894243 10.1192/bjp.2019.22

[37] Miglietta E, Belessiotis-Richards C, Ruggeri M, Priebe S (2018) Scales for assessing patient satisfaction with mental health care: a systematic review. J Psychiatr Res 100:33–46. 10.1016/j.jpsychires.2018.02.014 29482063 10.1016/j.jpsychires.2018.02.014

[38] Kuosmanen L, Hätönen H, Jyrkinen AR, Katajisto J, Välimäki M (2006) Patient satisfaction with psychiatric inpatient care. J Adv Nurs 55(6):655–663. 10.1111/j.1365-2648.2006.03957.x 16925614 10.1111/j.1365-2648.2006.03957.x

[39] Arnesen Y, Lillevoll KR, Mathiassen B (2023) User satisfaction in child and adolescent mental health service: comparison of background, clinical and service predictors for adolescent and parent satisfaction. Health Expectations 26(6):2608–2619. 10.1111/hex.13861 37650556 10.1111/hex.13861 PMC10632616

[40] Thapa Bajgain K, Amarbayan M, Wittevrongel K, McCabe E, Naqvi SF, Tang K, Aghajafari F, Zwicker JD, Santana M (2023) Patient-reported outcome measures used to improve youth mental health services: a systematic review. J Patient-reported Outcomes 7(1):14. 10.1186/s41687-023-00556-0 10.1186/s41687-023-00556-0 PMC9928989 36788182

[41] Bidonde J, Meneses-Echavez JF, Hafstad E, Brunborg GS, Bang L (2023) Methods, strategies, and incentives to increase response to mental health surveys among adolescents: a systematic review. BMC Med Res Method 23(1):270. 10.1186/s12874-023-02096-z 10.1186/s12874-023-02096-z PMC10652438 37974067

[42] Sjetne IS, Bjertnæs ØA, Olsen RV, Iversen HH, Bukholm G (2011) The generic short patient experiences questionnaire (GS-PEQ): identification of core items from a survey in Norway. BMC Health Serv Res 11:11. 10.1186/1472-6963-11-88 21510871 10.1186/1472-6963-11-88 PMC3111343

