# Correction: The outpatient experience questionnaire for child and adolescent mental health services: reliability and validity following a nationwide survey

**DOI:** 10.1186/s41687-025-00949-3

**Published:** 2025-09-03

**Authors:** Hilde Hestad Iversen, Mona Haugum, Oyvind Bjertnaes

**Affiliations:** Norwegian Institute of Public Health: Folkehelseinstituttet, PO Box 222, Skoyen, Oslo 0213 Norway

**Correction: Journal of Patient-Reported Outcomes 9, 20 (2025)**

10.1186/s41687-025-00852-x

Following publication of the original article it was reported that there was an error in Table 2. The following items were missing a double asterisk indication (**) indicating “Items finally selected for the short version of the instrument”:

- Item 5: Did your therapist seem to be good at their job?**
- Item 6: Did you feel that your therapist listened to what you had to say?**
- Item 8: Did you feel that your therapist cared about you?**
- Item 10: Were you able to talk to your therapist about things that were important to you?**
- Item 30: Overall, did you get the help you needed to better manage everyday life?**
- Item 31: Overall, did you get good help and treatment from CAMHS?**

The missing indication has been added to Table 2 and the original article has been updated.

